# Upregulation of Calcium Homeostasis Modulators in Contractile-To-Proliferative Phenotypical Transition of Pulmonary Arterial Smooth Muscle Cells

**DOI:** 10.3389/fphys.2021.714785

**Published:** 2021-08-02

**Authors:** Marisela Rodriguez, Jiyuan Chen, Pritesh P. Jain, Aleksandra Babicheva, Mingmei Xiong, Jifeng Li, Ning Lai, Tengteng Zhao, Moises Hernandez, Angela Balistrieri, Sophia Parmisano, Tatum Simonson, Ellen Breen, Daniela Valdez-Jasso, Patricia A. Thistlethwaite, John Y. -J. Shyy, Jian Wang, Joe G. N. Garcia, Ayako Makino, Jason X. -J. Yuan

**Affiliations:** ^1^Section of Physiology, Division of Pulmonary, Critical Care and Sleep Medicine, La Jolla, CA, United States; ^2^Department of Pediatrics, Tucson, AZ, United States; ^3^State Key Laboratory of Respiratory Diseases, First Affiliated Hospital of Guangzhou Medical University, Guangzhou, China; ^4^Beijing Chaoyang Hospital, Capital Medical University, Beijing, China; ^5^Division of Cardiothoracic Surgery, Department of Surgery, La Jolla, CA, United States; ^6^Department of Bioengineering, University of California, San Diego, La Jolla, CA, United States; ^7^Division of Cardiovascular Medicine, Department of Medicine, La Jolla, CA, United States; ^8^Department of Medicine, The University of Arizona, Tucson, AZ, United States; ^9^Division of Endocrinology and Metabolism, La Jolla, CA, United States

**Keywords:** smooth muscle cell, pulmonary hypertension, CALHM channels, contractile-to-proliferative, phenotypical transition

## Abstract

Excessive pulmonary artery (PA) smooth muscle cell (PASMC) proliferation and migration are implicated in the development of pathogenic pulmonary vascular remodeling characterized by concentric arterial wall thickening and arteriole muscularization in patients with pulmonary arterial hypertension (PAH). Pulmonary artery smooth muscle cell contractile-to-proliferative phenotypical transition is a process that promotes pulmonary vascular remodeling. A rise in cytosolic Ca^2+^ concentration [(Ca^2+^)_*cyt*_] in PASMCs is a trigger for pulmonary vasoconstriction and a stimulus for pulmonary vascular remodeling. Here, we report that the calcium homeostasis modulator (CALHM), a Ca^2+^ (and ATP) channel that is allosterically regulated by voltage and extracellular Ca^2+^, is upregulated during the PASMC contractile-to-proliferative phenotypical transition. Protein expression of CALHM1/2 in primary cultured PASMCs in media containing serum and growth factors (proliferative PASMC) was significantly greater than in freshly isolated PA (contractile PASMC) from the same rat. Upregulated CALHM1/2 in proliferative PASMCs were associated with an increased ratio of pAKT/AKT and pmTOR/mTOR and an increased expression of the cell proliferation marker PCNA, whereas serum starvation and rapamycin significantly downregulated CALHM1/2. Furthermore, CALHM1/2 were upregulated in freshly isolated PA from rats with monocrotaline (MCT)-induced PH and in primary cultured PASMC from patients with PAH in comparison to normal controls. Intraperitoneal injection of CGP 37157 (0.6 mg/kg, q8H), a non-selective blocker of CALHM channels, partially reversed established experimental PH. These data suggest that CALHM upregulation is involved in PASMC contractile-to-proliferative phenotypical transition. Ca^2+^ influx through upregulated CALHM1/2 may play an important role in the transition of sustained vasoconstriction to excessive vascular remodeling in PAH or precapillary PH. Calcium homeostasis modulator could potentially be a target to develop novel therapies for PAH.

## Introduction

Pulmonary arterial hypertension (PAH) is a fatal and progressive disease in which increased pulmonary arterial pressure (PAP) is mainly due to increased pulmonary vascular resistance (PVR) (Runo and Loyd, 2003). Elevated PAP and PVR result in an increase of afterload of the right ventricle (RV) which, if untreated, leads to right heart failure and death. Idiopathic PAH predominantly affects women (Farber et al., 2015; Batton et al., 2018). At the initial stage of the disease pathogenesis, sustained pulmonary vasoconstriction and attenuated pulmonary vasodilation may be the major cause for the elevated PVR and PAP in patients with PAH. As the disease progresses, patients may undergo a gradual transition from sustained vasoconstriction to relatively permanent structural changes of the pulmonary arteries such as concentric pulmonary vascular wall thickening (characterized by adventitial, medial and intimal hypertrophy) and muscularization of precapillary arterioles and capillaries (Ogata and Iijima, 1993; Solik et al., 2013; Jain M. et al., 2020). Similarly, neointimal and plexiform lesions in the pulmonary vasculature along with intraluminal occlusions of small pulmonary arteries and arterioles also contribute to the increase of PVR and PAP (Yu et al., 2004; Abe et al., 2010). Excessive pulmonary vascular remodeling in PAH is partially mediated by increased proliferation and migration of pulmonary arterial smooth muscle cells (PASMCs) due to PASMC dedifferentiation from a contractile or quiescent phenotype to a proliferative or synthetic phenotype (Beamish et al., 2010; Fernandez et al., 2015). The transition of PASMCs from a contractile to a proliferative phenotype is an important indicator of the progression of a pathological state of cardiovascular diseases such as atherosclerosis and PAH (Giannotti et al., 2019; Liu and Gomez, 2019; Zhang et al., 2020). Vascular smooth muscle cells (SMCs) or PASMCs and their role in the development of PAH require further investigation due to their phenotypic diversity which performs both contractile and synthetic or proliferative functions (Rensen et al., 2007).

Calcium homeostasis modulators (CALHM), including CALHM1 and CALHM2, are identified as a family of ion (cation and anion) channels and ATP channels in the plasma membrane that are sensitive to membrane voltage and extracellular Ca^2+^ level (Gallego-Sandin et al., 2011; Ma et al., 2012, 2016; Siebert et al., 2013; Demura et al., 2020; Ren et al., 2020). In PAH, a rise in cytosolic free Ca^2+^ concentration [(Ca^2+^)_*cyt*_] in PASMCs is not only a trigger for PASMC contraction and pulmonary vasoconstriction, but also a key stimulus for PASMC proliferation/migration and concentric pulmonary vascular remodeling (Fernandez et al., 2015; Song et al., 2018). CALHM1 and CALHM2 channels are closed at the resting membrane potential but can be opened by a strong membrane depolarization (Ma et al., 2016). The resting membrane potential in freshly dissociated contractile PASMCs (approximately −60 mV to −80 mV) or PASMCs in the intact PA (measured by intracellular recording) is often much more negative than that in primary cultured PASMCs in serum/growth factors-containing media (proliferative PASMC) (Yuan et al., 1993, Yuan, 1995, 1997; Seiden et al., 2000; Firth et al., 2011). The more depolarized proliferating PASMCs may require Ca^2+^ influx through CALHM1/2 to increase [Ca^2+^]_*cyt*_ that is required for or involved in propelling cells to go through the cell cycle and stimulating PASMC proliferation and maintaining a highly proliferative state. Reduction of extracellular [Ca^2+^] increases the probability for CALHM channels to open, which allows the channels to be activated at a negative potential. Similarly, due to the channels’ inability to discriminate between cations and anions, cations like Ca^2+^, anions like Cl^–^, and adenosine triphosphate (ATP) can all permeate as charged signaling ions and signaling molecules through the pore of CALHM, thereby contributing to the regulation of collective SMC functions such as contractile-to-proliferative phenotypic transition (Rensen et al., 2007). Ultimately, it is known that the increased [Ca^2+^]_*cyt*_ due to upregulated and activated Ca^2+^-permeable cation channels contributes to excessive proliferation of PASMCs and concentric pulmonary vascular remodeling in patients with PAH and in animals with experimental PH (Firth et al., 2013).

A rise in [Ca^2+^]_*cyt*_ due to upregulated and activated Ca^2+^ channels such as CALHM1/2 channels in PASMCs plays an important role in the development and progression of concentric pulmonary remodeling and muscularization of precapillary arterioles in PAH. In this study, we sought to study whether CALHM1 and/or CALHM2 were involved in PASMC phenotypical transition from the contractile or differentiated phenotype to the synthetic or proliferative phenotype, and whether CALHM1/2 are upregulated in PASMCs from patients with PAH and animals with experimental PH in comparison to respective controls.

## Materials and Methods

### *In vivo* Animal Experiments

All experimental procedures on animals were approved by the Institutional Animal Care and Use Committee (IACUC) of the University of California, San Diego (UCSD) (S18205 for mice and S18211 for rats). Standardized protocols were followed for conducting the rodent model of experiments. For the establishment of monocrotaline (MCT)-induced PH in rats, we randomly divided Sprague-Dawley rats (males, 6–8 weeks old) into two groups: a control group and an MCT-injected group. In the MCT group, a single subcutaneous injection of MCT (60 mg/kg, Sigma Aldrich) was given at day 1 to establish the MCT-induced PH. In control rats, a single subcutaneous injection of vehicle control was used. MCT solution was prepared by dissolving MCT in 0.5N HCl to 200 mg/ml that was neutralized with 0.5-N NaOH to pH 7.4 and further diluted with sterile water to obtain a final concentration of 60 mg/ml. All animals were housed in the UCSD Animal Research Facility. Food and water were provided *ad libitum*. Approximately 28 days after MCT injection, the rats were used for hemodynamic measurement and for phenotypic characterization studies. To measure pulmonary hemodynamics in rats, a Millar catheter (SPR-847 1.4F) was inserted into the RV via the external right jugular vein for live monitoring of right ventricular pressure (RVP). Given the fact that systolic RVP or right ventriculary systolic pressure (RVSP) equals to pulmonary arterial systolic pressure, we used RVSP as a surrogate to indicate pulmonary arterial systolic pressure in rodents. RVP values were recorded and analyzed by the Millar MPVS Ultra and PowerLab 8/30 data acquisition system (AD Instruments). After hemodynamic measurement, the whole-heart was excised, dissected and measured to calculate the Fulton Index.

To establish chronic hypoxia-induced PH (HPH) in mice for the pharmacological experiments, animals (approximately 25g body weight, male, 8 weeks old) were exposed to normoxia (room air) or normobaric hypoxia (10% O_2_) in a well-ventilated chamber for 6 weeks. Then, mice were intraperitoneally injected (i.p.) with DMSO (vehicle) or CGP-37157 (0.6 mg/kg) every 8 h (q8H) for two weeks under the hypoxic conditions to see whether CGP-37157 attenuates established PH. The total duration of hypoxic exposure was 8 weeks: first 6 weeks to establish HPH and then 2 weeks, during hypoxic exposure, for the CGP-37157 treatment. Then RVP and RV contractility (RV- ± dP/dt) were measured in the mice by right heart catheterization using a pressure catheter (Millar Instruments, PVR1030, 1F, 4E, 3mm, 4.5cm, Colorado, United States) introduced via the external right jugular vein as previously described (Tang et al., 2018a; Jain P. P. et al., 2020). The mean pulmonary arterial pressure (mPAP) was calculated using the formula: mPAP = 0.61 × RVSP+2 (in mmHg), where RVSP is the value of right ventricular systolic pressure. Data were recorded and analyzed using Lab Chart Pro1.0 software (AD Instruments). After hemodynamic measurements, mice were euthanized by pentobarbital (120 mg/kg, i.p.) and phenotypic characterization studies were performed.

### *Ex vivo* Lung Angiogram

Lung angiogram was used to estimate pulmonary vascular density and remodeling in mice as described previously (Refs, Smith, Jain). Briefly, after right heart catheterization measuring hemodynamics, heparin was first injected into the RV to prevent coagulation in the pulmonary circulation. Then, a polyethylene tube was inserted into the main PA and PBS was superfused through the tube to washout residual blood and clots in the pulmonary vasculature. Then, Microfil (MV-122, Flow-Tech, Inc., Carver, MA), as a casting polymer, was superfused into PA using a small pump (Farmingdale, New York, United States) at a speed of 0.05 ml/min for 1–2 min until the whole lungs were filled with the casting polymer. The casting polymer-filled lungs were then isolated and stored overnight at 4°C. Twenty-four hrs later, the polymer-filled lungs were dehydrated by immersing in ethanol (one hour each in 50%, 70%, 80%, 95%, and 100%, and one more hour in 100%) to show the vascular tree. The dehydrated lungs with polymers filled in the vasculature were immersed into methyl salicylate solution, placed on a shaker overnight, and then photographed with a digital camera through a dissecting microscope. The peripheral area of the lung vascular images from apical, middle and basal regions was selected using Photoshop software and converted to binary images using NIH ImageJ software to quantify the total length of lung vascular branches (mm/mm^2^, the number of vascular branches (mm^–2^) and the number of vascular branch junctions (mm^–2^) at a given area (mm^2^).

### Assessment of RV Hypertrophy

The heart was dissected from the animal first, and then both right and left atria were removed. The RV was carefully separated from the remaining heart tissue that includes the left ventricle (LV) and the septum (S). The RV and the LV+S tissues were immediatedly weighed after dissection to avoid the effect of tissue dehydration on weight. The ratio of the weight of the RV to the weight of the LV and S [RV/(LV+S)], also referred to as Fulton Index, was then calculated and used to assess RV hypertrophy (Wang et al., 2004).

### Isolation of Intrapulmonary Arterial Segments and Preparation of Primary Cultured PASMC

Isolation of rat PA was performed as previously described (Fernandez et al., 2015). Briefly, the isolated lungs and heart were placed in Hanks Balanced Salt Solution (HBSS) supplemented with 10 mM HEPES. The main PA was first isolated and dissected from the heart followed by further isolation of the left and right branches of main PA and the intrapulmonary arterial branches under the magnified microscope. Then the isolated PA was cleaned by removing fat and connective tissues and incubated in HBSS with 1.7 mg/mL collagenase type II for 20 min at 37°C. The shortly digested PA ring was rinsed with HBSS to remove residual collagenase. Finally, the adventitia was carefully removed with fine forceps and the endothelium was scrapped off with a surgical scalpel under microscope. The remaining PA, mainly PA smooth muscle tissue (or fresly isolated PA), was used as contractile PASMC for extracting total protein and RNA for the proposed experiment.

The PA smooth muscle tissue from the same rat was then used to prepare primary cultured PASMCs. Briefly, the PA smooth muscle segment was digested in HBSS supplemented with 1.7 mg/ml collagenase (type II), 0.5 mg/ml elastase (Sigma, Cat. #: E7885), and 1 mg/ml of bovine serum albumin (BSA, Sigma, Cat. #: A3059-100G) at 37°C for 50 min followed by gentle trituration with a Pasteur pipette to dissociate the cells. The digestion was then stopped by adding 10 ml of Dulbecco’s modified Eagle’s medium (DMEM) with 20% fetal bovine serum (FBS). The cell suspension was centrifuged, and the supernatant was aspirated off. The pellet was re-suspended in 2 ml of fresh 10% FBS DMEM and triturated for a couple of times to further separate the cells. Freshly dissociated PASMCs were then plated directly onto 100-mm Petri dishes in 10% FBS DMEM. The cells were cultured in a humidified 5% CO_2_ incubator at 37°C. The medium was changed to smooth muscle growth medium (Lifeline Cell Technologies, Cat. #: LL-0014) 24 h after initial seeding and was changed every 48 h subsequently. The primary cultured PASMCs (incubated in the serum- and growth factor-containing smooth muscle growth medium) from the same rat were used as synthetic or proliferative PASMC for the proposed experiments.

### Immunostaining

Primary cultured rat PASMCs were fixed in 4% paraformaldehyde at +4°C for 10 min. Cells were then washed twice with PBS and permeabilized with 0.5% Triton X-100 in PBS for 10 min. Unspecific binding was reduced by blocking with 5% BSA (Sigma) in PBS for 60 min. Specimens were then incubated with primary antibody ACTA2 (Abcam, Cat.#: ab220179) at +4°C overnight, then with Alexa Flour 488 (anti-mouse)-conjugated secondary antibody at room temperature for 2 h. Nuclei were stained with DAPI. Fluorescent images were acquired using an Olympus FV100 confocal microscope.

### Western Blotting

Whole-lung tissue, freshly isolated PA and primary cultured PASMCs were lysed with cold RIPA lysis buffer (ThermoFisher Scientific, Cat. #: 89901) supplemented with protease inhibitor cocktail (Roche, Cat.#: 11836153001) followed by incubation on ice for 15 min and centrifugation at 13,300 rpm for 15 min at +4°C. The pellet was discarded, and the supernatant was used to measure the protein concentration. Protein samples were loaded to SDS-PAGE and transferred onto 0.45 μm nitrocellulose membranes. Then the membranes were blocked in 0.1% Tween 20 in 1X TBS (TBST) with 5% nonfat dry milk powder for 1 h at room temperature followed by the incubation with primary antibodies in 5% BSA-TBST on the rocker overnight at +4°C. On the next day, membranes were washed and incubated with secondary antibody for 1 h at room temperature in TBST containing 5% milk. Finally, membranes were washed, and the blots were developed with enhanced chemiluminescence substrate (Pierce, Rockford, IL). Primary antibodies used in this study included anti-MHC11 (Santa Cruz Biotechnology, Cat. #: sc-376157), anti-TAGLN (Abcam, Cat. #: Ab14106), anti-ACTA2 (Abcam, Cat. #: Ab7817), anti-VIM (Santa Cruz Biotechnology, Cat. #: sc-5565), anti-PCNA (Santa Cruz Biotechnology, Cat. #: sc-25280), anti-PDGF-AA (Santa Cruz Biotechnology, Cat. #: sc-9974), anti-Actin (Cell Signaling Technology, Cat. #: 4968), anti-CALHM1 (Abcam, Cat. #: ab106561), anti-CALHM2 (Abcam, Cat. #: ab121446), anti-AKT (Cell Signaling Technology, Cat. #: 4691), anti-pAKT (Cell Signaling Technology, Cat. #: 4060), anti-mTOR (Cell Signaling Technology, Cat. #: 2983), anti-pmTOR (Cell Signaling Technology, Cat. #: 2971), anti-GAPDH (Abcam, Cat. #: Ab8245), anti-PDGFRα (Cell Signaling Technology, Cat. #: 3174) and anti-β-actin (Santa Cruz Biotechnology, Cat. #: sc-47778). Secondary antibodies included anti-mouse (Cell Signaling Technology, Cat. #: 7076S) or anti-rabbit (Cell Signaling, Cat. #: 7074S). Band intensity was quantified with ImageJ and normalized to pan-actin or GAPDH as a loading control.

### Human Smooth Muscle Cell Culture

The approval for the use of human PASMCs was granted by the University of California, San Diego Institutional Review Board (IRB, #200396X). Pulmonary arterial smooth muscle cells isolated from 6 healthy subjects and 6 IPAH patients provided by the Pulmonary Hypertension Breakthrough Initiative (PHBI) were used for this study. Purity of PASMCs in culture was confirmed by FACS and ICC using α-SMA, SM22α, and SM-MHC with 95% of positive cells. The human PASMCs were cultured in 100-mm Petri dishes in smooth muscle growth medium in a humidified 5% CO_2_ at 37°C. The fresh medium was changed 24 h after initial seeding and every 48 h subsequently. Cells were allowed to grow to 80–90% confluence and harvested for protein isolation.

### Cell Proliferation Assay

To determine PASMC proliferation, we measured changes of the number of viable cells before (0 h) and after (48 h) incubation of cells in media containing 10% FBS. Human PASMCs were first evenly dispensed in 96-well plate containing 10% FBS media. When the cells grew to approximately 60% confluence, the growth medium containing 10% FBS was changed to 0.3% FBS medium (for 24 h) to synchronize the cells in the G_0_/G_1_ phase. The synchronized cells were split into two groups, vehicle (DMSO) control group and CGP 37157 (100 nM)-treated group, and incubated in 5% FBS media with vehicle or CGP for 48 h. Then, 10 μl CCK8 solution (Cell Counting Kit-8, Cat. No. K1018, ApexBio, Houston, TX) was added to in each well of the two groups of cells and incubated for 2 h. Absorbance at 450 nm was then measured using a microplate reader (iMark, Bio-Rad Laboratories, Hercules, CA) to indicate the number of viable cells. For the siRNA experiments, synchronized cells were transfected with a control siRNA (siNT) and siRNA for CALHM1 (siCALHM1, Cat. #, sc-90815, Santa Cruz Biotechnology, TX) or siRNA for CALHM2 (siCALHM2, Cat. #, sc-90780, Santa Cruz Biotechnology) in 10% FBS media (for 72 h) using RNAiMax transfection reagent (Cat. #, 13778075, Invitrogen). Forty-eight hours later, CCK8 solution was added into the cell wells and incubated for 2 h, and then number of viable cells was determined by measuring absorbance at 450 nm in each group of cells.

### Drugs and Chemicals

CGP-37157 (MedChemExpress, Cat.#: HY-15754) or rapamycin (Cayman Chemical, Cat.#: 13346) were dissolved in DMSO as a stock solution. The stock solution was then aliquoted and kept frozen at −20°C until further use. Rat PASMC were treated with vehicle or rapamycin (10 nM) in basal SMC medium without any FBS or growth factors for 24 h.

### Statistical Analysis

Data are expressed as means ± standard error (SE) with the number (*n*) of individual biological replicates. Statistical significance was analyzed by Student’s *t*-test (paired or unpaired as applicable) between two groups or by ANOVA among many groups using SigmaPlot software. A value *p* < 0.05 was accepted as statistically significant. Significant difference is expressed in the figures or figure legends as ^∗^*p* < 0.05 and ^#^*p* < 0.05.

## Results

**FIGURE 1:**
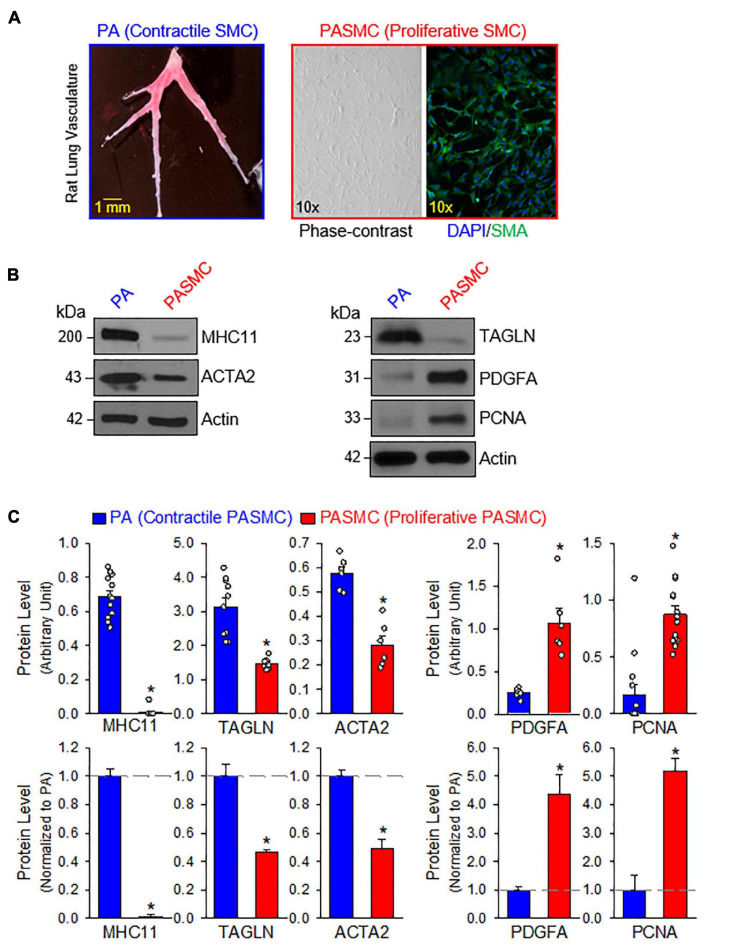
Contractile and proliferative phenotypes of pulmonary arterial smooth muscle cells (PASMCs). **(A)**: Pulmonary artery (PA) branches isolated from the left lobe of rat lung (left panel). Primary cultured PASMCs (derived from the same rat) in medium containing serum and growth factors (right panels), including a phase-contrast image and a fluorescent image showing DAPI (4′,6-diamidino-2-phenylindole, in blue) and α smooth muscle actin (SMA, in red). **(B)**: Western blot analyses on markers of contractile SMC, myosin heavy chain 11 (MHC11), SM22-α or transgelin (TAGLN) and SMα2-actin (ACTA2) as well as markers of synthetic/proliferative PASMC, platelet-derived growth factor A (PDGFA) and proliferation cell nuclear antigen (PCNA) in freshly isolated PA (mainly containing contractile PASMC) and primary cultured PASMCs (mainly containing proliferative PASMCs). **(C)**: Summarized data (mean ± SE, *n* = 6–14 in each group) showing protein expression levels of MHC11, TAGLN, ACTA2, PDGFA, and PCNA in freshly isolated PA [PA (Contractile PASMC)] and primary cultured PASMCs [PASMC (Proliferative PASMC)]. Data are presented as arbitrary units (upper panels) or normalized to PA tissue (lower panels). The horizontal broken lines in the lower panels indicate the level of the proteins in the PA. *p < 0.05 vs. PA (Contractile PASMC).

The contractile-to-proliferative transition of PASMCs was examined by comparing freshly isolated PA tissue, representative of the contractile PASMC phenotype, and primary cultured PASMC, representative of the proliferative (or synthetic) phenotype prepared from the same rats. To confirm that PASMC underwent the phenotypical switch from the contractile state to proliferative (synthetic) state in our model, we used Western blot analysis to compare markers specific for the contractile and proliferative phenotypes. Then, we used the same model to compare the expression of target proteins between the freshly isolated PA with the endothelium denuded and adventitia stripped-off (Figure 1A, left panel), which contain mainly contractile PASMC, and the primary cultured PASMCs (prepared from the same PA segments) in media contained 10% FBS and growth factors (Figure 1A, right panels), which contained mainly proliferative PASMCs. The purity of primary cultured PASMCs was confirmed by co-staining cells with SMA and DAPI (Figure 1A, right panel).

We first conducted a series of Western blot experiments on the markers representing contractile phenotype and proliferative phenotype of SMCs. As shown in Figures 1B,C, the freshly isolated PA highly expressed the markers for contractile SMC including myosin heavy chain 11 (MHC11), transgelin (TAGLN, or SM22-α) and smooth muscle α2 actin (ACTA2); while the primary cultured PASMCs in media containing serum and growth factors highly expressed platelet-derived growth factor A (PDGFA) and the marker for cell proliferation, proliferation cell nuclear antigen (PCNA). Western blot analyses showed that protein expression levels of the contractile markers (e.g., MHC11, TAGLN, and ACTA2) were significantly higher in freshly isolated PA than in primary cultured PASMCs, while protein expression levels of the synthetic markers (e.g., PDGF-A) and proliferation marker (i.e., PCNA) were significantly higher in primary cultured PASMCs than in freshly isolated PA (Figure 1C). These results indicate that using freshly isolated PA and primary cultured PASMCs (prepared from the same rat) is a good model to study the differences between contractile and proliferative phenotypes of PASMC.

### CALHM Is Upregulated in Proliferative PASMC Compared to Contractile PASMCs

**FIGURE 2:**
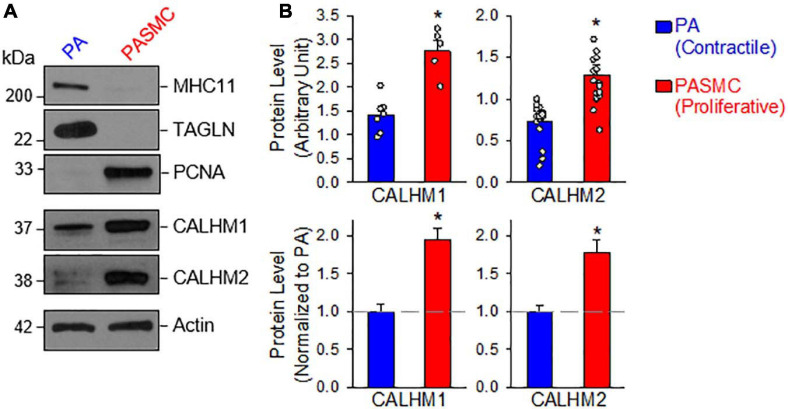
Upregulated calcium homeostasis modulator (CALHM1 and CALHM2) protein expression in proliferative PASMCs. **(A)**: Western blot analyses on MHC11, TAGLN, and PCNA as well as CALHM1 and CALHM2 in freshly isolated PA (mainly containing contractile PASMCs) and primary cultured PASMCs (mainly containing proliferative PASMCs). **(B)**: Summarized data (mean ± SE) showing protein expression levels of CALHM1 (*n* = 7) and CALHM2 (*n* = 17) in the PA and in PASMCs. Data are presented as arbitrary units (upper panels) or normalized to PA tissue (lower panels). The horizontal broken lines indicate the level of the CALHM1/2 proteins in the PA. *p < 0.05 vs. PA (Contractile PASMC).

Next, we aimed to determine and compare the protein expression of CALHM1 and CALHM2 in contractile and proliferative PASMCs using the same model of freshly isolated PA (highly expressed MHC11 and TAGLN) and primary cultured PASMCs (highly expressed PCNA) from the same rat (Figure 2A). The protein levels of CALHM1 and CALHM2 were significantly higher in primary cultured PASMCs than in freshly isolated PA (Figures 2A,B). The expression levels of CALHM1/2 were inversely proportional to the expression levels of the contractile markers, MHC11 and TAGLN, but directly proportional to the expression level of the proliferation marker, PCNA (Figure 2). These results indicate that the upregulation of CALHM1 and CALHM2 is associated with the contractile-to-proliferative phenotypical transition of PASMCs.

### Activation of AKT/mTOR Signaling Is Involved in Contractile-to-Proliferative Phenotypical Transition of PASMCs

**FIGURE 3:**
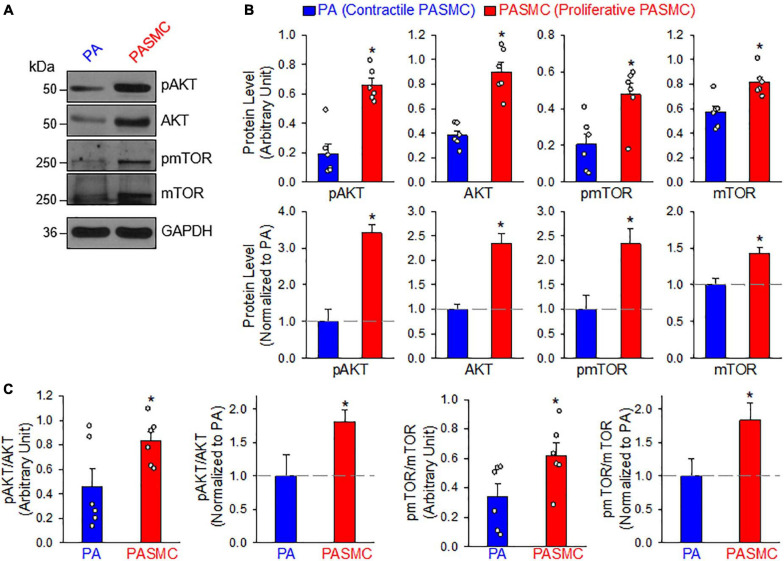
AKT/mTOR signaling is increased in synthetic or proliferative PASMCs. **(A)**: Representative Western blot analyses on phosphorylated (p) AKT (pAKT), AKT, pmTOR, and mTOR in freshly isolated PA (mainly containing contractile PASMCs) and primary cultured PASMCs (mainly containing proliferative PASMC). GAPDH was used as loading control. **(B)**: Summarized data (mean ± SE, *n* = 6) showing protein expression levels of pAKT, AKT, pmTOR, and mTOR in the PA and PASMCs. Data are presented as arbitrary units (upper panels) or normalized to PA tissue (lower panels). **(C)**: Summarized data (mean ± SE, *n* = 6) showing the ratio of pAKT/AKT and pmTOR/mTOR in the PA and the PASMCs. The horizontal broken lines indicate the protein level in the PA. *p < 0.05 vs. PA (Contractile PASMC).

The PI3K/AKT/mTOR signaling cascade is a major signaling pathway involved in cell proliferation and protein synthesis in a variety of cell types including cancer cells and PASMCs (Goncharov et al., 2014; Sahoo et al., 2016; Meng et al., 2017; Babicheva et al., 2021). Our lab and other investigators have reported that the PI3K/AKT1/mTORC1/C2 pathways are involved in the development and progression of experimental PH in mice (Tang et al., 2015, 2018b; Goncharova et al., 2020). The next set of experiments was designed to investigate whether the transition of PASMCs from the contractile (isolated PA) to proliferative (primary cultured PASMC) phenotype requires upregulation or activation of the signaling proteins involved in the PI3K/AKT/mTOR pathway. As shown in Figure 3, the PASMC phenotypical transition was also associated with a significant upregulation of AKT and mTOR (Figures 3A,B) and a significant increase in phosphorylation of AKT (pAKT) and mTOR (pmTOR) (Figure 3C). The upregulated AKT/mTOR and enhanced pAKT/pmTOR in proliferative PASMCs were proportionally correlated to the proliferation maker, PCNA, and were inversely proportional to the contractile markers, MHC11, ACTA2, and TAGLN (see Figure 1). These data indicate that the contractile-to-proliferative phenotypical transition of PASMCs requires or involves upregulation (AKT and mTOR) and activation (pAKT and pmTOR) of the AKT/mTOR signaling pathway. Enhanced PI3K/AKT/mTOR signaling pathway is an important signaling cascade in PASMC proliferation and, potentially, the development and progression of pulmonary vascular remodeling in patients with PAH and animals with experimental PH.

### Inhibition of AKT/mTOR Signaling Downregulates CALHM Expression in Proliferative PASMCs

**FIGURE 4:**
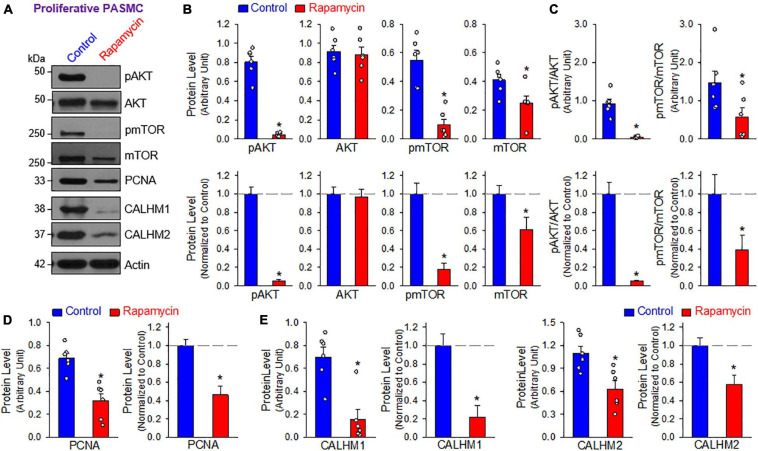
Inhibition of mTOR with rapamycin downregulates protein expression of CALHM1/2 in proliferating PASMCs. **(A)**: Representative Western blot analyses on phosphorylated (p) AKT (pAKT), AKT, pmTOR, mTOR, and PCNA as well as CALHM1 and CALHM2 in primary cultured PASMCs treated with vehicle (Control) or rapamycin (Rapamycin, 10 nM) for 24 h. **(B)**: Summarized data (mean ± SE, *n* = 5–6 experiments) showing levels of pAKT, AKT, pmTOR, and mTOR in control and rapamycin-treated proliferating PASMCs. **(C)**: Summarized data (mean ± SE, *n* = 6 experiments) showing the ratio of pAKT/AKT and pmTOR/mTOR in control and rapamycin-treated proliferating PASMCs. **(D)**: Summarized data (mean ± SE, *n* = 6) showing the level of PCNA, a cell proliferation marker, in control and rapamycin-treated proliferating PASMCs. **(E)**: Summarized data (mean ± SE, *n* = 6) showing protein expression levels of CALHM1 and CALHM2 in control and rapamycin-treated proliferating PASMCs. Data are presented as arbitrary units as well as normalized to PA tissue. *p < 0.05 vs. PA (Contractile PASMC).

**FIGURE 5:**
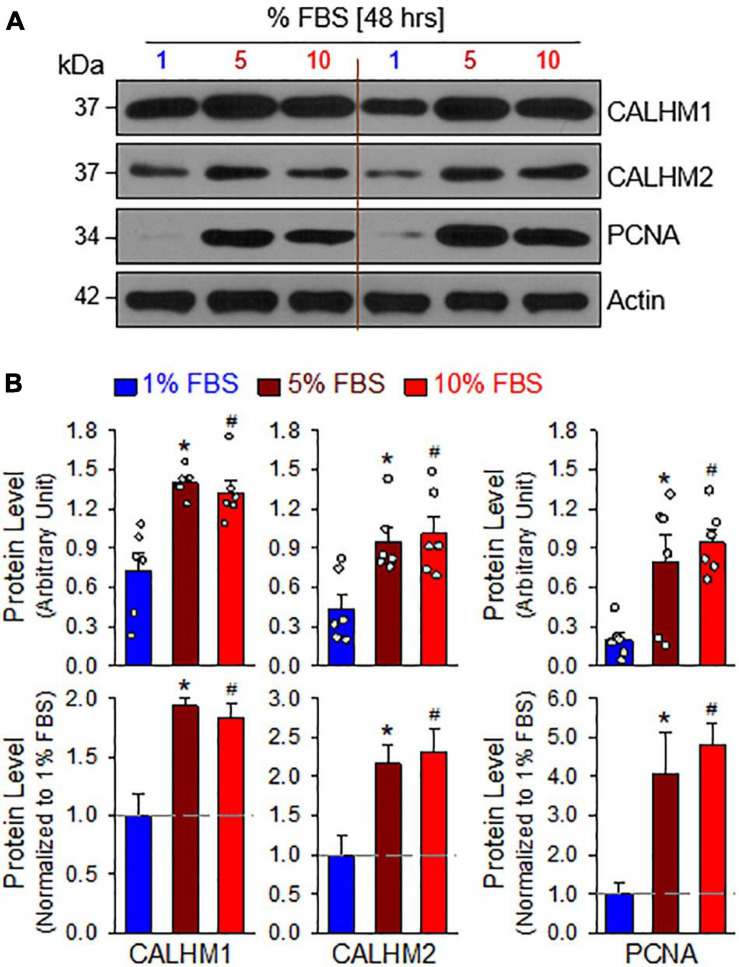
Serum starvation inhibits PASMC proliferation and downregulates protein expression of CALHM1/2 in proliferating PASMCs. **(A)**: Representative Western blot analyses on CALHM1, CALHM2 and PCNA in primary cultured PASMCs incubated in culture media containing 1%, 5%, and 10% fetal bovine serum (FBS, for 48 h). **(B)**: Summarized data (mean ± SE, *n* = 6 experiments) showing levels of CALHM1, CALHM2, and PCNA in PASMCs cultured in 1%, 5%, and 10% FBS-containing media. *p < 0.05 and ^#^*p* < 0.05 vs. PASMC cultured in 1% FBS medium.

The mTOR complex 1 (mTORC1) and mTORC2 play important roles in the regulation of cell proliferation and protein expression, while both complexes are involved in the development and progression of PH (Houssaini et al., 2013, 2016; Goncharov et al., 2014; Lee et al., 2016; Guo et al., 2019; Goncharova et al., 2020; Babicheva et al., 2021). PASMCs in the normal PA exhibit a differentiated, quiescent, and contractile phenotype. Imbalanced release of various growth and fibrotic factors from circulating and inflammatory cells results in PASMC transition from the contractile phenotype to the synthetic or proliferative phenotype. The mTORC1 activity plays an important role in regulating phenotypical transitions of vascular SMCs (Owens et al., 2004; Rzucidlo et al., 2007; Liu and Gomez, 2019). Activation of mTORC1 by platelet-derived growth factor-BB (PDGF-BB) promotes the transition of SMCs from a contractile to a proliferative phenotype, whereas inhibition of mTORC1 with rapamycin induces SMC differentiation and inhibits SMC proliferation (Rzucidlo et al., 2007; Majed and Khalil, 2012). Treatment of primary cultured proliferative PASMCs with rapamycin (10 nM for 24 h), an inhibitor of mTOR, significantly decreased the pAKT and mTOR (pmTOR) (Figures 4A,B), and the ratio of pAKT/AKT and pmTOR/mTOR (Figure 4C). The rapamycin-associated inhibition of AKT/mTOR signaling was correlated with a significant decrease in the proliferative cell marker PCNA (Figures 4A,D) and a marked downregulation of CALHM1/2 (Figures 4A,E). In addition to the inhibitory effect of rapamycin on CALHM1/2, we also examined whether serum starvation had a similar effect on CALHM1/2 expression in primary cultured proliferative PASMCs. As shown in Figure 5, reducing serum (FBS) in the culture media from 10 to 1% significantly downregulated protein expression of CALHM1 and CALHM2, while the downregulation of CALHM1/2 (Figure 5B) was closely associated with the inhibition of PASMC proliferation, indicated by the PCNA (Figure 5C). These data indicate that mTORC1/C2 activity and expression level of CALHM1/2 are required for, or involved in, PASMC proliferation, and likely the development and progression of pulmonary vascular remodeling in patients with PAH and animals with experimental PH.

### CALHM Is Increased During the PASMC Phenotypical Transition in Rats With MCT-Induced PH

**FIGURE 6:**
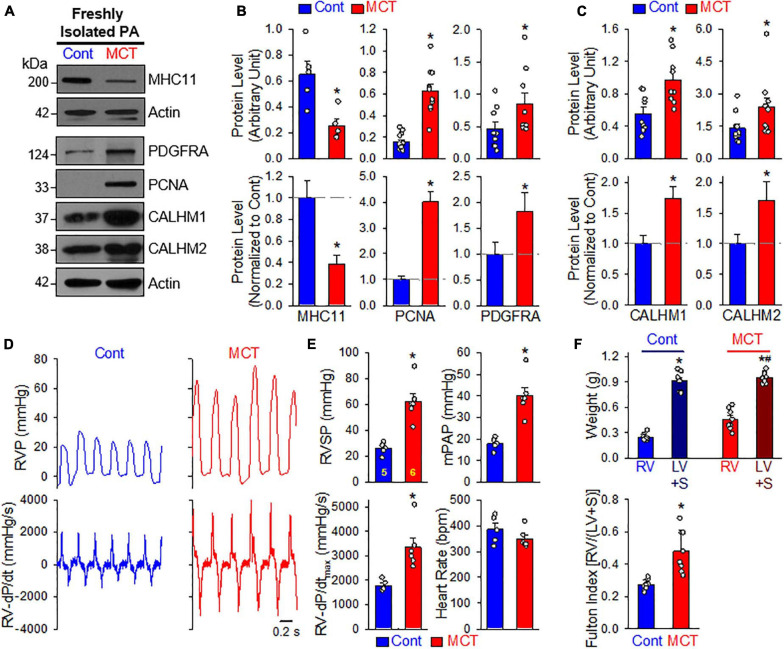
Upregulated CALHM1 and CALHM2 in freshly isolated PA from rats with monocrotaline (MCT)-induced pulmonary hypertension (PH). **(A–C)**: Representative images **(A)** and summarized data (mean ± SE, *n* = 5–13) showing protein levels of MHC11 (*n* = 5), PCNA (*n* = 13), PDGF receptor α (PDGFRα, *n* = 9) **(B)** as well as CALHM1 and CALHM2 **(C)** in freshly isolated PA (mainly containing contractile PASMC) from vehicle- (Cont) or MCT-injected rats. *p < 0.05 vs. Cont rats. **(D)**: Representative records of right ventricular pressure (RVP, upper panels) and right ventricule (RV) contractility (RV- ± dP/dt, lower panels) in control rats (Cont) and MCT-injected rats (MCT). **(E)**: Summarized data (mean ± SE, *n* = 5 for Cont rats and *n* = 6 for MCT rats) showing right ventricular systolic pressure (RVSP, a surrogate measure of pulmonary arterial systolic pressure), mean PAP (mPAP), RV-+dP/dt_*max*_ and heart rate in control rats and MCT-injected rats. *p < 0.05 vs. Cont rats. **(F)**: Summarized data (mean ± SE, *n* = 9 for each group) showing the dry weight of the RV and the left ventricle (LV) and septum (S) (upper panel), as well as Fulton Index (lower panel), the ratio of the weight of RV to the weight of LV and S [RV/(LV+S)] in vehicle- (Cont) or MCT-injected rats. *p < 0.05 vs. RV in Control rats; ^#^*p* < 0.05 vs. RV in MCT-injected rats (upper panel); *p < 0.05 vs. Cont rats (lower panel).

In the next set of experiments, we developed an MCT-induced PH model (MCT-PH) in rats to determine whether PASMC underwent the contractile-to-proliferative phenotypical transition in this animal model. At first, we compared the protein expressions of the contractile marker MHC11 and the synthetic/proliferative marker PDGF receptor α (PDGFRα or PDGFRA), as well as the proliferation marker PCNA in PA tissue isolated from control and MCT-PH rats. Western blot analyses showed that the protein level of MHC11 was significantly lower while protein levels of PDGFRα and PCNA were significantly higher in freshly isolated PA from MCT-PH rats compared with control rats (Figures 6A,B). Next, we measured protein expression level of CALHM1 and CALHM2 in the control and MCT-PH rats. As expected, CALHM1/2 were significantly upregulated in freshly isolated PA from MCT-PH rats in comparison to the PA from normal controls (Figures 6A,C). The upregulated expression of CALHM1/2 in the PA from MCT-PH rats was inversely proportional to the expression level of the contractile marker MHC11, but was positively correlated to the proliferative markers PCNA and PDGFRα (Figures 6A–C). To confirm the successful development of MCT-PH in rats, we measured RVP and RV contractility (RV- ± dP/dt) by right heart catheterization and assessed RV hypertrophy using the Fulton index as an indicator. MCT injection (60 mg/kg, once at the beginning of experiments) significantly increased RVSP, a surrogate measure for pulmonary arterial systolic pressure, mean PAP (mPAP, which was calculated by the equation: mPAP = 0.61RVSP + 2), and RV-dP/dt_*max*_ 4 weeks after initial MCT injection (Figures 6D,E). The Fulton index, or the ratio of the weight of the RV to the weight of the LV and S [RV/(LV+S)], also significantly increased 4 weeks after MCT injection (Figure 6F). The hemodynamic data and Fulton Index both indicated the PH phenotype in MCT-injected rats. These results imply that PASMCs may undergo a potential contractile-to-proliferative transition during the development of MCT-PH, while CALHM1/2 are upregulated during the transition either as a cause or a consequence of the phenotypical transition of PASMCs in MCT-PH rats.

### CALHM Is Upregulated in PASMCs Isolated From PAH Patients

**FIGURE 7:**
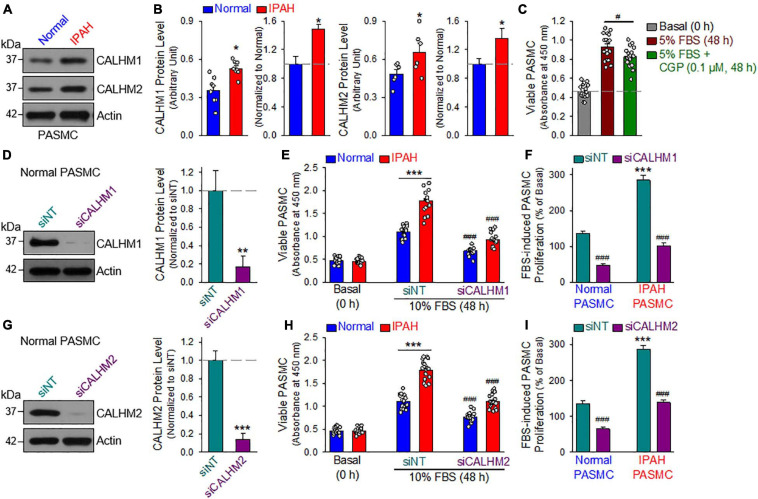
Upregulated CALHM1/2 in proliferating PASMCs from patients with idiopathic pulmonary arterial hypertension (IPAH) and inhibitor or downregulation of CALHM1/2 significantly inhibits PASMC proliferation. **(A,B)**: Representative images **(A)** and summarized data (mean ± SE, **B**) showing protein level of CALHM1 (*n* = 8) and CALHM2 (*n* = 6) in primary cultured PASMCs isolated from normal subjects (Normol) and patients with IPAH. **p < 0.01 vs. Normol PASMC. **(C)**: Summarized data (mean ± SE, *n* = 18) showing number of viable PASMCs (determined by absorbance at 450 nm) before (Basal, 0 h) and after 48 h of incubation in 5% FBS media in the absence (5% FBS) and presence of CGP 37157 (CGP, 0.1 μM, 48 h) (5% FBS + CGP). *p < 0.01 between 5% FBS and 5% FBS+CGP. **(D)**: Representative image (left) and summarized data (mean ± SE, *n* = 6, right) showing Western blot analyses on CALHM1 in normal PASMC treated with control siRNA (siNT) and siRNA for CALHM1 (siCALHM1). **p < 0.01 vs. siNT. **(E)**: Summarized data (mean ± SE, *n* = 18) showing number of Normal PASMCs and IPAH PASMCs before (Basal, 0 h) and 48 h after incubation in 10% FBS media in the presence of control siRNA (siNT) and siRNA for CALHM1 (siCALHM1). ***p < 0.001 between Normal and IPAH; ^###^*p* < 0.001 vs. siNT. **(F)**: 10% FBS (48 h)-mediated increases in viable PASMC numbers (or 10% FBS-induced PASMC proliferation, % of the basal cell number at 0 h) in normal and IPAH PASMCs treated with siNT and siCALHM1. Data are constructed from the number of viable cells shown in panel E. ***p < 0.001 vs. Normal PASMC; ^###^*p* < 0.001 vs. siNT. **(G)**: Representative image (left) and summarized data (mean ± SE, *n* = 6, right) showing Western blot analyses on CALHM2 in normal PASMC treated with siNT and siRNA for CALHM2 (siCALHM2). ***p < 0.01 vs. siNT. **(H)**: Summarized data (mean ± SE, *n* = 18) showing number of normal and IPAH PASMCs before (Basal, 0 h) and 48 h after incubation in 10% FBS media containing siNT and siCALHM2. ***p < 0.001 between Normal and IPAH PASMC; ^###^*p* < 0.001 vs. siNT. **(I)**: 10% FBS (48 h)-mediated increases in viable PASMC numbers (or 10% FBS-induced PASMC proliferation, % of the basal cell number at 0 h) in normal and IPAH PASMCs treated with siNT and siCALHM2. Data are constructed from the number of viable cells shown in panel H. ***p < 0.001 vs. Normal PASMC; ^###^*p* < 0.001 vs. siNT.

Furthermore, we also measured CALHM1/2 protein levels in PASMCs from normal subjects and patients with idiopathic PAH (IPAH). As shown in Figures 7A,B, the protein expression levels of CALHM1 and CALHM2 in proliferating PASMCs from patients with IPAH were significantly higher than in cells from normal subjects. These results suggest that upregulated CALHM1/2, or enhanced Ca^2+^ influx through CALHM1/2 (Ma et al., 2012, 2016) and increased ATP release through CALHM1/2 (Siebert et al., 2013; Jun et al., 2018), is involved in the development and progression of concentric pulmonary arterial wall thickening due to excessive PASMC proliferation and migration. Indeed, inhibition of CALHM1 with CGP 37157, a benzothiazepine that has been demonstrated to Ca^2+^ influx through CALHM1 (Moreno-Ortega et al., 2015; Garrosa et al., 2020), significantly inhibited serum (FBS)-mediated proliferation in normal human PASMC (Figure 7C). To confirm the potential role of CALHM1 and CALHM2 in PASMC proliferation, we used siRNA to downregulate CALHM1 and CALHM2 (Figures 7D,G). Specific downregulation of CALHM1 (Figures 7E,F) or CALHM2 (Figures 7H,I) with siRNA significantly inhibited 10% FBS-mediated PASMC proliferation in cells from normal subjects and patients with IPAH. As noted, the increase in viable cells induced by 40 hrs of incubation in media containing 10% FBS in IPAH PASMC was significantly greater than in normal PASMC (Figures 7E,F,H,I); however, the siRNA-mediated inhibition was comparable in normal PASMC (65.4 ± 15.6% and 51.9 ± 16.6% decreases by siRNA for CALHM1 and CALHM2, respectively) (Figure 7F) and IPAH PASMC (64.4 ± 11.1% and 51.7 ± 11.1% decreases by siRNA for CALHM1 and CALHM2, respectively) (Figure 7I). These data indicate that CALHM1/2 are involved in PASMC proliferation, while blockade of CALHM1/2 can inhibit PASMC proliferation.

### Inhibition of CALHM Reverses Experimental PH

**FIGURE 8:**
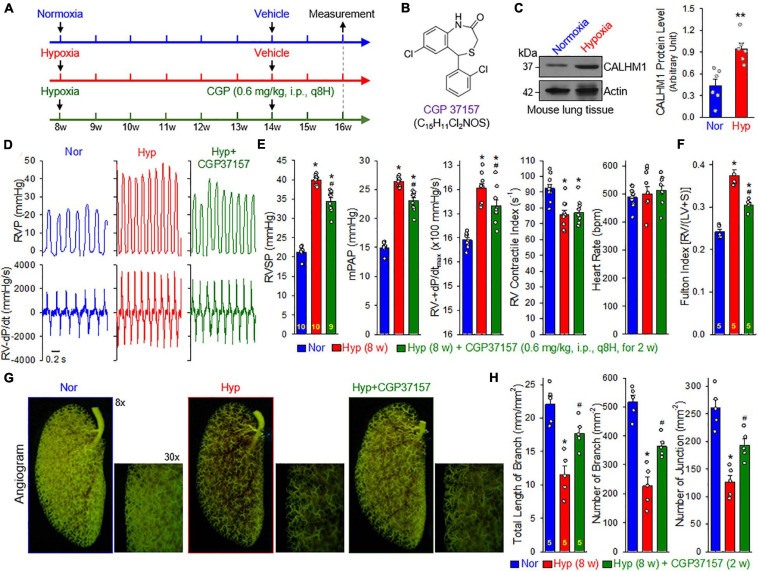
Blockade of CALHM ion channels with CGP 37157 partially reverses established experimental pulmonary hypertension (PH) in mice. **(A)**: Experimental protocol for the reversal experiment using CGP 37157 (CGP). Eight-week-old C57Bl/6 mice were subjected to normoxia (Nor) or normobaric hypoxia (10% O_2_) for 6 weeks at first. CGP 37157 (0.6 mg/kg, q8H) was then intraperitoneally administered (i.p.) for two more weeks during hypoxia. **(B)**: Chemical structure of CGP 37157. **(C)**: Representative images (left panels) and summarized data (mean ± SE, *n* = 6, right panel) showing Western blot analysis on CALHM1 in the whole-lung tissues of normoxic control mice (Normoxia or Nor) and chronically hypoxic mice (Hypoxia or Hyp). **p < 0.01 vs. Nor. **(D)**: Representative records of right ventricular pressure (RVP) (upper panels) and right ventricle (RV) contractility (RV- ± dP/dt) (lower panels) in normoxic (Nor) control mice and hypoxic mice receiving vehicle (Hyp) or CGP37157 (0.6 mg/kg, i.p., q8H for 2 wks.) (Hyp+CGP37157). **(E)**: Summarized data (mean ± SE, *n* = 9–10 per group) showing right ventricular systolic pressure (RVSP), estimated mean pulmonary arterial pressure (mPAP), RV-+dP/dt_*max*_ and heart rate in Nor, Hyp, and Hyp+CGP mice. *p < 0.05 vs. Normoxia (Nor); ^#^*p* < 0.05 vs. Hyp (ANOVA). **(F)**: Summarized data (means ± SE, *n* = 5 per group) showing the Fulton Index, the ratio of the weight of right ventricle (RV) to the weight of left ventricle (LV) and septum (S) [RV/(LV+S)] in Nor, Hyp, and Hyp+CGP mice. *p < 0.05 vs. Normoxia (Nor); ^#^*p* < 0.05 vs. Hyp (ANOVA). **(G)**: Representative images of angiograph of the left lung at 8× and 30× magnification from Nor, Hyp, and Hyp+CGP37257 (0.6 mg/kg, i.p., q8H for 2 wks.) mice. Images (30×) are enlarged images from the selected areas of the 8× images. **(H)**: Summarized data (mean ± SE, *n* = 5 in each group) showing the total length of lung vascular branches (left panel), the number of lung vascular branches (middle panel) and the number of vascular branch junctions (right panel) per square millimeter of the selected lung area from Nor, Hyp, and Hyp+CPG37157 mice. *p < 0.05 vs. Nor; ^#^*p* < 0.05 vs. Hyp (ANOVA).

To examine whether pharmacological blockade of CALHM channels is able to reverse or inhibit experimental PH, we conducted an *in vivo* reversal experiment in mice with established experimental PH using CGP 37157, a non-selective blocker of CALHM1 channels (Figure 8A). CGP 37157 or 7-chloro-5-(2-chlorophenyl)-1,5-dihydro-4,1-benzothiazepin-2(3H)-one (C_15_H_11_Cl_2_NOS) (Figure 8B) is a benzothiazepine that has been demonstrated to be a selective inhibitor of the mitochondrial Na^+^/Ca^2+^ exchanger (IC_50_ = 0.36–5 μM for different cell types) (Cox et al., 1993; Parnis et al., 2013), and a blocker of Ca^2+^ influx through CALHM1 (Moreno-Ortega et al., 2015; Garrosa et al., 2020). In whole lung tissues, we found that CALHM1 protein expression was significantly increased in mice with chronic hypoxia-induced PH (Figure 8C). After 8 weeks of hypoxia exposure (10% O_2_), mice successfully developed PH (Figure 8D) indicated by significantly increased RVSP, a surrogate measure for pulmonary arterial systolic pressure) (Figures 8D,E), increased mean PAP (mPAP, calculated by the equation, mPAP = 0.61RVSP+2, based on RVSP), (Chemla et al., 2004; Parasuraman et al., 2016) increased RV contractility (RV− ± dP/dt_*max*_) as a result of increased RVSP (Figures 8D,E) and RV hypertrophy (Figure 8F). Intraperitoneal injection (i.p.) of CGP 37157 every 8 hrs a day (q8H) for two weeks partially reversed established PH (Figures 8D–F): CGP 37157 resulted in a 29.4 ± 4.9% inhibition of increased RVSP (from 18.7 ± 0.4 to 13.2 ± 0.9, *n* = 9; *p* < 0.05), a 29.4 ± 4.9% inhibition of increased mPAP (from 11.4 ± 0.2 to 8.1 ± 0.6, *n* = 9; *p* < 0.05), a 34.8 ± 11.5% inhibition of increased RV-+dP/dt_*max*_ (from 1078.1 ± 90.6 to 703.0 ± 124.5, *n* = 9; *p* < 0.05), and a 52.0 ± 4.6% inhibition of RV hypertrophy or Fulton Index (from 0.134 ± 0.014 to 0.064 ± 0.006, *n* = 5; *p* < 0.05). CGP 37157 had negligible effects on RV contractile index and heart rate (Figure 8E).

Furthermore, we conducted an angiogram experiment to determine whether CGP 37157 reversed pulmonary vascular remodeling in mice with established PH (Figure 8G). Exposure of the animals to normobaric hypoxia (Hyp, 10% O_2_) for 8 w significantly decreased the total length of lung vascular branches (from 22.0 ± 1.3 to 11.5 ± 1.4 mm/mm^2^, *n* = 5; *p* < 0.05), the number of lung vascular branches (from 516.7 ± 25.6 to 226.4 ± 31.0 per mm^2^, *n* = 5; *p* < 0.05) and the number of lung vascular branch junctions (from 260.2 ± 15.7 to 126.6 ± 11.8 per mm^2^, *n* = 5; *p* < 0.05). Intraperitoneal injection of CGP 37157 (0.6 mg/kg, q8H for 2 w) partially restored the total length of branches (from 11.5 ± 1.4 to 17.7 ± 1.0 mm/mm^2^, *n* = 5; *p* < 0.05), the number of branches (from 226.4 ± 31.0 to 364.1 ± 16.3 mm^–2^, *n* = 5; *p* < 0.05) and the number of junctions (from 126.6 ± 11.8 to 192.6 ± 11.8 mm^–2^, *n* = 5; *p* < 0.05) (Figure 8H). The angiography data are consistent with the hemodynamic data showing that CGP 37157, by blocking CALHM channels, significantly inhibits hypoxia-mediated increases in PAP and hypoxia-induced pulmonary vascular remodeling. Taken together with the hemodynamic data, these findings indicate that CALHM channels contribute to the development of experimental PH and that CALHM channels may be a therapeutic target to develop novel therapies for PAH and PH due to lung diseases and/or hypoxemia.

## Discussion

Transition or switch of vascular SMCs from the contractile (or full differentiated) phenotype to the synthetic or proliferative phenotype is implicated in many cardiovascular diseases (Owens et al., 2004; Rzucidlo et al., 2007; Gomez and Owens, 2012; Alencar et al., 2020; Nagao et al., 2020). In this study, we first used an ex vivo model, freshly isolated PA (containing mainly contractile PASMCs) and primary cultured PASMCs in media containing 10% FBS and growth factors (containing mainly proliferative PASMCs), to identify the membrane receptors, ion channels and intracellular signaling proteins that are associated with the PASMC phenotypic transition from a contractile phenotype to a proliferative phenotype. Our results indicate that (*i*) CALHM1 and CALHM2, two voltage-activated and extracellular Ca^2+^-inhibited Ca^2+^ channels and ATP release channels, are upregulated during the contractile-to-proliferative phenotypical transition in PASMCs; (*ii*) upregulated CALHM1/2 are associated with upregulated AKT and mTOR as well as an increased pAKT/AKT and pmTOR/mTOR ratio in proliferative PASMC compared to contractile PASMCs; (*iii*) inhibition of AKT/mTOR signaling with rapamycin (which disrupts the function of both mTORC1 and mTORC2) decreases the ratio of pAKT/AKT and pmTOR/mTOR and downregulates CALHM1/2 in proliferative PASMCs; (*iv*) serum starvation (decreasing serum from 10 to 1%) decreases PCNA, a marker of cell proliferation, and downregulates CALHM1/2 in proliferative PASMCs; (*v*) the protein expression level of CALHM1/2 in freshly isolated PA (mainly containing contractile PASMC) from animals with experimental PH and in primary cultured PASMC (mainly containing proliferative PASMC) from patients with idiopathic PAH is greater than in those from control animals and normal subjects; and (*vi*) pharmacological blockade of CALHM1 with CGP 37157 (0.6 mg/kg, i.p., q8H for 2 w) partially reverses established experimental PH. The observations from this study suggest that the PI3K/AKT/mTORC1 signaling cascade and the upregulated CALHM are either involved in or significantly influenced by PASMC contractile-to-proliferative phenotypical transition. Ca^2+^ influx through upregulated CALHM1/2 is probably required for or involved in stimulating and maintaining proliferation or overgrowth of PASMCs contributing to the development and progression of pulmonary vascular remodeling in PAH and PH.

Abnormal PASMC contraction (causing sustained vasoconstriction), migration and proliferation (contributing to vascular medial hypertrophy and occlusive lesions) have been implicated in the development and progression of PAH due to PASMCs’ phenotypic diversity (e.g., contractile and synthetic functions). The phenotypic diversity of SMCs has been studied as a function of collective gene programming, biochemical factors, extracellular matrix components and physical factors such as stretch and shear stress (Humbert et al., 2004; Rensen et al., 2007; Hassoun et al., 2009; Morrell et al., 2009; Kuhr et al., 2012; Tuder et al., 2013). Another way of defining phenotypical change is cell differentiation, which is defined as the process by which multipotential cells in the developing organism acquire cell-specific characteristics that distinguish them from other cell types. Three major regulatory components have been suggested in vascular SMC multifunctional development: *a*) selective activation of the subset of genes required for the cell’s differentiated functions; *b*) the coordinate control of expression of cell selective and/or specific genes at precise times and stoichiometry; and *c*) continuous regulation of gene expression through effects of local environmental cues on the genetic program that determines cell lineage, which includes the control of chromatin structure or epigenetic programming that can influence the ability of transcription factors to access the regulatory regions of genes (Owens et al., 2004).

In adult animals and normal subjects, vascular SMCs are specialized cells that contribute to vasoconstriction and vasodilation regulating blood pressure and blood flow distribution (Owens et al., 2004). Differentiated SMCs in adult blood vessels proliferate at an extremely low rate, exhibit low synthetic activity, and express a collection of contractile proteins, ion channels, and signaling molecules required for the cell’s contractile function (Owens, 1995; Somlyo and Somlyo, 2000; Rzucidlo et al., 2007; Alexander and Owens, 2012). Vascular SMCs, including PASMCs, present within the systemic and pulmonary arterial wall are highly sensitive to mechanical stress, humoral stimuli and local vasoactive substances (Lacolley et al., 2012). A phenotypic switch of PASMCs from a contractile to proliferative phenotype is inevitable for the pathological vascular remodeling process to occur. In healthy arteries, SMCs are surrounded by a basement membrane composed of laminin, collagen type IV, and heparin sulfate proteoglycan (Jain M. et al., 2020). In the normal PA media, PASMCs express a variety of “SMC markers” including SMC myosin heavy chain (MHC11), smooth muscle 22α (TAGLN22), SMC α2 actin (ACTA2), smoothelin and others (Bennett et al., 2016; Chakraborty et al., 2019), all of which are important factors of vascular SMCs’ ECM integrity and contractile function. During the development of intimal hyperplasia (IH), for example, and other vascular pathologies, such as restenosis and atherosclerosis, SMCs lose their contractile proteins and cellular quiescence, while increasing their migration, proliferation, and production of ECM proteins (Beamish et al., 2010). These processes define a “pathogenic” shift from a normal and contractile SMC/PASMC phenotype toward a synthetic or proliferative phenotype (Beamish et al., 2009, 2010). Smooth muscle cell phenotypical transition or SMC proliferation following arterial injury or inflammation is associated with hypertrophic inward remodeling of resistance arteries characterized by reduction in lumen diameter and in an increased media to lumen ratio. The transformation of SMCs from the contractile to the synthetic proliferative state is an important indicator toward the progression of a pathological state in systemic and pulmonary hypertension. The PASMC contractile-to-proliferative switch and resultant increase in PASMC migration and proliferation also promote PASMC transfer into the intima forming occlusive intimal lesions (Morrell et al., 2009; Tuder et al., 2013; Humbert et al., 2019) and the distal arterioles causing muscularization of precapillary arterioles and capillaries (Sheikh et al., 2015, 2018; Ntokou et al., 2021). One of the key pathological mechanisms involved in PASMC contractile-to-proliferative transition and PASMC proliferation in PAH is increased synthesis and release of PDGF from lung tissue (Yi et al., 1996, 2000; Sakao and Tatsumi, 2011; Batton et al., 2018). PDGF drives pulmonary vascular wall hypertrophy as it is significantly increased in lung tissue from PAH patient in comparison to control lungs, similar to many other mitogenic and inflammatory factors (TGF-β1, TLR-4, IL-1β, IL-18, etc.) (Raines and Ferri, 2005; Schermuly et al., 2005; Batton et al., 2018). PDGF-mediated PASMC proliferation involves an increase in cytosolic free Ca^2+^ concentration [Ca^2+^]_*cyt*_ which is also a key stimulus for this phenotypic transition (Morrell et al., 2009; Batton et al., 2018).

Calcium homeostasis modulator 1 (CALHM1), formerly known as FAM26C (Ma et al., 2016), is an important plasma membrane ion channel that is permeable to cations and anions. CALHM is regulated by membrane voltage and extracellular Ca^2+^ concentration. Through convergent evolution, CALHM has structural features similar to connexins, pannexins, and innexins (Siebert et al., 2013) which include four transmembrane helices with cytoplasmic amino and carboxyl termini, based on membrane topology prediction algorithms (Demura et al., 2020; Ren et al., 2020). Human CALHM1 is predicted to be a membrane protein with 346 amino acids. The functional CALHM1 channel is a hexamer (two CALHM1 octamers) containing a functional pore with a diameter of ∼14 Å (Ma et al., 2016; Demura et al., 2020; Ren et al., 2020). CALHM1 enables both cations and anions to go through due to its inability to discriminate between the two charged molecules. In addition, Ca^2+^ and ATP can both permeate through CALHM as signaling molecules through its pore. In the presence of physiological Ca^2+^, CALHM is closed at membrane resting potentials but can be opened by strong depolarization. Thus, reducing extracellular Ca^2+^ increases channel opening probability, enabling channel activation at negative membrane potentials (Ma et al., 2016). CALHM1 localizes to the plasma membrane and the sarcoplasmic/endoplasmic reticulum (SR/ER). In addition to mediating Ca^2+^ influx, CALHM1 may contribute to Ca^2+^ leak from the SR/ER to the cytosol (Gallego-Sandin et al., 2011). Increased Ca^2+^ leak through CALHM1 or CALHM2 in the SR/ER and inhibition of Ca^2+^ uptake into the SR/ER (Gallego-Sandin et al., 2011) can lead to store depletion and store-operated Ca^2+^ entry, another important pathway to increase [Ca^2+^]_*cyt*_ required for or involved in PASMC proliferation and migration and pulmonary vascular remodeling in PAH/PH (Sweeney et al., 2002; Yu et al., 2003; Song et al., 2018).

Five homologs of CALHM are identified in humans. The CALHM gene family, CALHM1 and its homologues are collectively identified as the FAM26 gene family, where six genes are found in two clusters on two chromosomes. CALHM1 (FAM26C) is clustered on chromosome 10 with the CALHM3 (FAM26A) and CALHM2 (FAM26B) genes. The FAM26D, FAM26E, and FAM26F genes, of which CALHM names have not been assigned, are located in a cluster on chromosome 6. All of the CALHM genes and previously named FAM26 genes, are present throughout vertebrates and conserved across more than 20 species including mouse, human, and *C.* elegans. (Ma et al., 2016) Co-expression of CALHM1 with CALHM3, but not with CALHM2, drastically enhances the activation kinetics of currents compared to expression of CALHM1 alone (Oka, 2018; Sclafani and Ackroff, 2018). CALHM1 and CALHM2 may thus form homomeric and heteromeric channels in native smooth muscle cells. CALHM expression led to a robust and relatively selective activation of the Ca^2+^-sensitive kinases, such as MEK1/2, ERK1/2, RSK1/2/3, and MSK1. CALHM1-mediated activation of ERK1/2 signaling, or Ca^2+^ activation of ERK1/2 signaling by Ca^2+^ influx through CALHM1 in the plasma membrane and Ca^2+^ mobilization through CALHM1 in the SR/ER, plays an important role in the regulation of cell proliferation and gene expression (Morrell et al., 2009) and the development of pulmonary vascular remodeling in patients with PAH and animals with experimental PH (Awad et al., 2016). In addition, Ca^2+^-sensitive signaling pathways including ERK, c-Jun-N-terminal kinase, p38 mitogen activated protein kinases, Akt, Rho/Rho-kinase, and calcineurin and calmodulin kinases have been associated with cofactors and transcription factors that are in part mediated by transcription repression in phenotypic modulation of vascular SMCs (Owens et al., 2004; Lacolley et al., 2012). In addition to its permeability to Ca^2+^, CALHM1 has been identified as a novel ATP-permeable channel that mediates the action potential-dependent release of ATP or action potential-dependent fast purinergic neurotransmission (Taruno, 2018). Intracellular concentration of ATP [(ATP)_*i*_] is around millimolar (mM) range, while extracellular ATP concentration is very low. Given the report that CALHM1/2 are also ATP channels allowing outward transportation of ATP from the cytosol to the extracellular or intercellular sites, upregulated CALHM1/2 may also be involved in enhancing ATP-mediated mitogenic and migratory effects on PASMC via activation of purinergic receptors (P2Y and P2X) in the plasma membrane. Metabolic shift and abnormalities have been implicated in the development and progression of pulmonary vascular remodeling, a major cause for the elevated pulmonary vascular resistance and pulmonary arterial pressure in patients with PAH and animals with experimental PH. Upregulated CALHM1/2 in proliferative PASMCs and in PA from animals with experimental PH may be a potential link between intracellularly accumulated ATP and extracellular ATP-mediated PASMC proliferation and migration and, ultimately, pulmonary vascular remodeling. Furthermore, ATP released from lung vascular endothelial cells through CALHM1/2 channels can also serve as an important ligand to stimulate PASMC proliferation and migration through activation of purinergic receptors (P2Y and P2X) in PAH and PH (Visovatti et al., 2016; Hennigs et al., 2019; Strassheim et al., 2020).

Although we focused on the role of CALHM1/2 in the contractile-to-proliferative phenotypical transition and proliferation of PASMC in this study, we also found that CALHM1/2 were expressed in freshly isolated PA tissues (mainly containing contractile PASMC) so Ca^2+^ influx through CALHM1/2 should be involved in the regulation of PASMC contraction and migration. Rare variants in CALHM1 are also found to be associated with early onset of Alzheimer’s disease by regulating Ca^2+^ homeostasis (Rubio-Moscardo et al., 2013). Increased vascular stiffness is one of the characteristics in vascular aging, abnormal expression and function of CALHM1 may be involved in the regulation of vascular stiffness. Furthermore, CALHM1 also allows intracellular ATP to exit cell, while extracellular ATP plays an important role in the regulation of vasoconstriction and vasodilation (Mortensen et al., 2009; Lang et al., 2017; Cai et al., 2020). More studies are needed to define the functional role of CALHM1 and CALHM2 in sustained pulmonary vasoconstriction and vascular stiffen in the development of PAH and PH due to respiratory diseases and/or hypoxia.

For the *in vivo* pharmacological experiments, we used CGP 37157, a benzothiazepine that has been demonstrated to block CALHM1 channels (Moreno-Ortega et al., 2015; Garrosa et al., 2020). CGP 37157 inhibited Ca^2+^ influx through CALHM1 channels at low concentrations in neurons; 0.1-10 μM of CGP 37157 resulted in 50% inhibition of Ca^2+^ influx through CALHM1 in neurons and neuroblastoma cells (Moreno-Ortega et al., 2015). In neuroblastoma cells, 20-30 μM CALHM1 significantly inhibited toxicity effect induced by Ca^2+^ influx through CALHM channels by 80% (Gonzalez-Lafuente et al., 2012). Pharmacological blockade of CALHM1 with CGP 37157 (10 μM) also resulted in more than 50% inhibition of oxygen glucose deprivation-mediated toxic effect on hippocampus neurons due to neuronal Ca^2+^ overload through CALHM1. CGP 37157 has, however, long been demonstrated as an inhibitor of the mitochondrial Na^+^/Ca^2+^ exchanger (NCX) with an IC_50_ = 0.36–5 μM for different cell types (Cox et al., 1993; Parnis et al., 2013). The inhibitory effects of CGP 37157 on CALHM channels and mitochondrial NCX are difficult to differentiate solely based on the dose or concentration (Cox et al., 1993; Parnis et al., 2013; Moreno-Ortega et al., 2015; Garrosa et al., 2020). Our *in vitro* experiments showed that a low dose (100 nM) of CGP 37157 significantly inhibited FBS-mediated human PASMC proliferation, suggesting that the subthreshold dose of CGP 37157 for mitochondrial NCX was sufficient to inhibit PASMC proliferation by, possibly, blocking CALHM1 channels. We previously reported that the plasmalemmal NCX was upregulated and inward transportation of Ca^2+^ via NCX was enhanced in PASMCs from patients with PAH and animals with experimental PH (Zhang et al., 2005, 2007). Our *in vitro* and *in vivo* experimental data, however, cannot rule out the possibility that the inhibitory effect of CGP 37157 on PASMC proliferation and therapeutic or reversal effect of CGP 37157 on experimental PH are, at least partially, due to its blocking effect on the mitochondrial NCX. The [Ca^2+^] in mitochondrial matrix plays an important role in ATP production, while Ca^2+^ efflux from the mitochondrial matrix to the cytosol is regulated by the mitochondrial NCX (Boyman et al., 2013). Acute treatment of rat PASMCs with the mitochondrial uncoupler, FCCP, caused a rapid increase in [Ca^2+^]_*cyt*_ due to Ca^2+^ mobilization from mitochondria to the cytosol that was sufficient to activate Ca^2+^-activated K^+^ channels in the plasma membrane; while long time treatment with FCCP in the presence of Ca^2+^ chelators inhibited voltage-gated K^+^ channels in the plasma membrane (Yuan et al., 1996). These studies indicated that the mitochondrial [Ca^2+^] and cytosolic [Ca^2+^] functionally communicate or cooperate with each other via Ca^2+^ transporters in the mitochondrial membrane (e.g., the mitochondrial Ca^2+^ uniporter and NCX) to regulate cardiac and smooth muscle cell contraction, migration, proliferation and apoptosis (Yuan et al., 1996; Poburko et al., 2006; Boyman et al., 2013). More studies are needed to investigate whether CGP 37157 also inhibits NCX in the plasma membrane in human and animal PASMCs and whether mitochondrial NCX in PASMCs is involved in the development of experimental PH.

The gender differences in terms of disease severity and mortality as well as drug response in patients with idiopathic PAH and animals with experimental PH have been demonstrated in many studies (Mair et al., 2014; Batton et al., 2018; Morris et al., 2021). One of the limitations in our *in vivo* animal experiments was that we only used male animals. It is important and interesting to study sex-associated expression and function of CALHM1 and CALHM2 in pulmonary vascular smooth muscle cells and endothelial cells. Furthermore, we will seek for new drugs that are more selective and specific to block CALHM1 or CALHM2 to conduct prevention and reversal pharmacological experiments in both female and male animals.

In summary, the observations from this study suggest that upregulation of CALHM1/2 is involved in the PASMC contractile-to-proliferative phenotypical switch due to increased Ca^2+^ influx through CALHM and enhanced ATP release from the cytosol to extracellular matrix through upregulated CALHM. The increased cytosolic [Ca^2+^] due to Ca^2+^ influx through CALHM1/2 and increased extracellular [ATP] due to ATP transportation through upregulated CALHM1/2 stimulate PASMC proliferation contributing to the development and progression of pulmonary vascular remodeling in patients with PAH and animals with experimental PH. Indeed, blockade of CALHM channels with a non-selective inhibitor, CGP 37157 PG, partially reverses established experimental PH in animals. CALHM1/2 are potentially new targets to develop novel therapies for PAH and precapillary PH.

## Data Availability Statement

The raw data supporting the conclusions of this article will be made available by the authors, without undue reservation.

## Ethics Statement

The animal study was reviewed and approved by the Institutional Animal Care and Use Committee (IACUC) of the University of California, San Diego (UCSD).

## Author Contributions

JY initiated the project and designed the study. MR wrote the initial draft of the manuscript. MR and JC performed molecular functional experiments and analyzed data. JC and PJ performed animal experiments and analyzed data. ABab, MX, JL, NL, TZ, MH, ABal, and SP contributed to editing the manuscript. TS, EB, DV-J, PT, JS, JW, JG, and AM participated in the discussion on experimental design and critically reviewed the manuscript. All authors contributed to the article and approved the submitted version.

## Conflict of Interest

The handling editor declared a shared affiliation with one of the author MR at the time of the review. The remaining authors declare that the research was conducted in the absence of any commercial or financial relationships that could be construed as a potential conflict of interest.

## Publisher’s Note

All claims expressed in this article are solely those of the authors and do not necessarily represent those of their affiliated organizations, or those of the publisher, the editors and the reviewers. Any product that may be evaluated in this article, or claim that may be made by its manufacturer, is not guaranteed or endorsed by the publisher.
