# Supplementary figures and images for: Upregulation of Calcium Homeostasis Modulators in Contractile-To-Proliferative Phenotypical Transition of Pulmonary Arterial Smooth Muscle Cells
Source: Front Physiol. 2021 Aug 2;12:714785. doi: 10.3389/fphys.2021.714785 (PMC8364962; doi:10.3389/fphys.2021.714785)

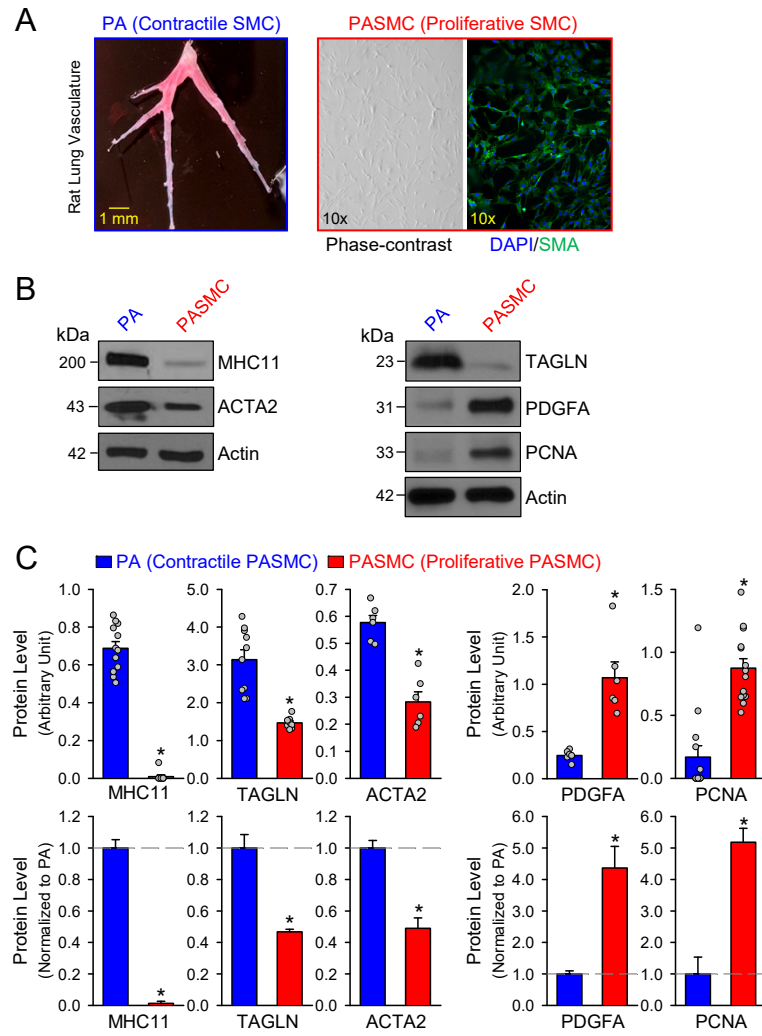

Figure 1

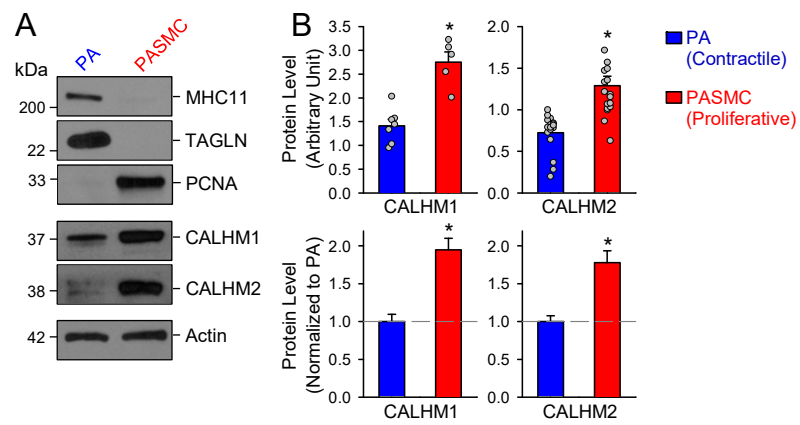

Figure 2

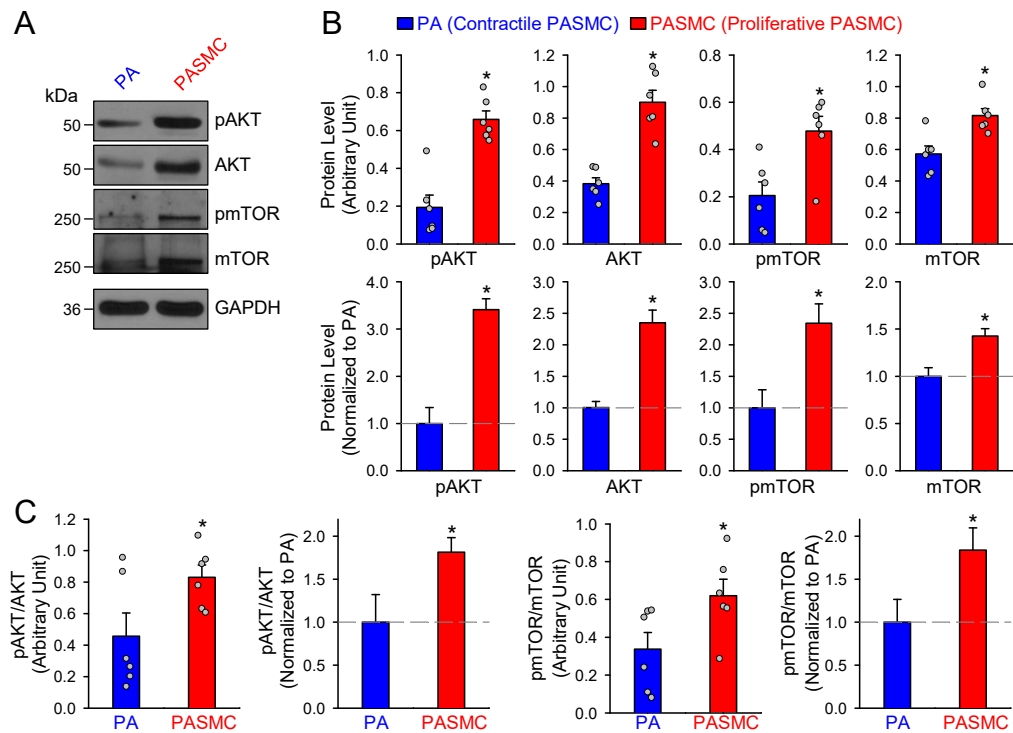

Figure 3

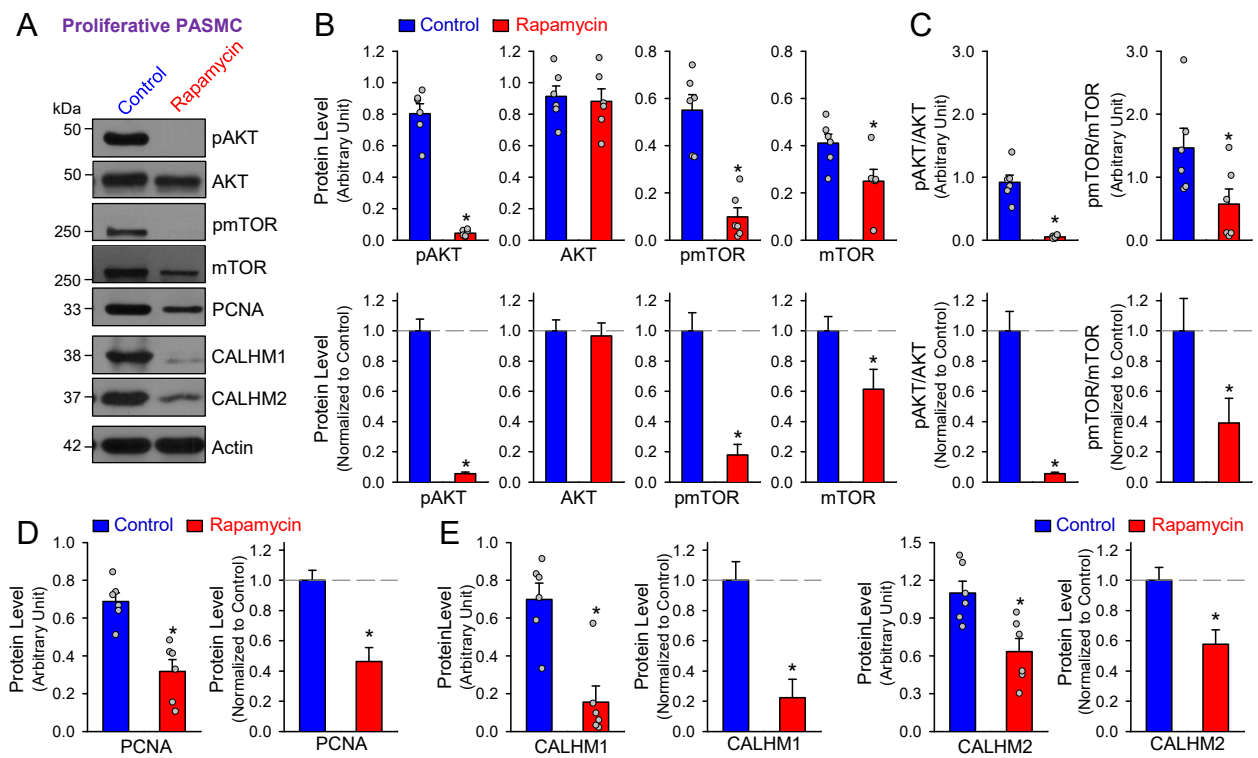

Figure 4

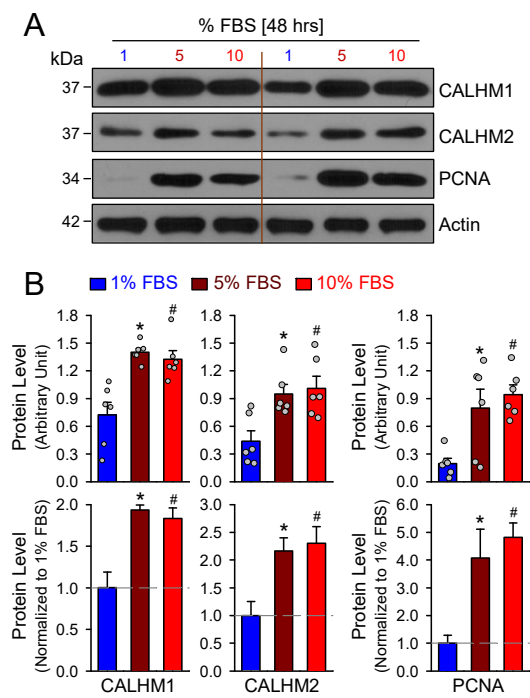

Figure 5

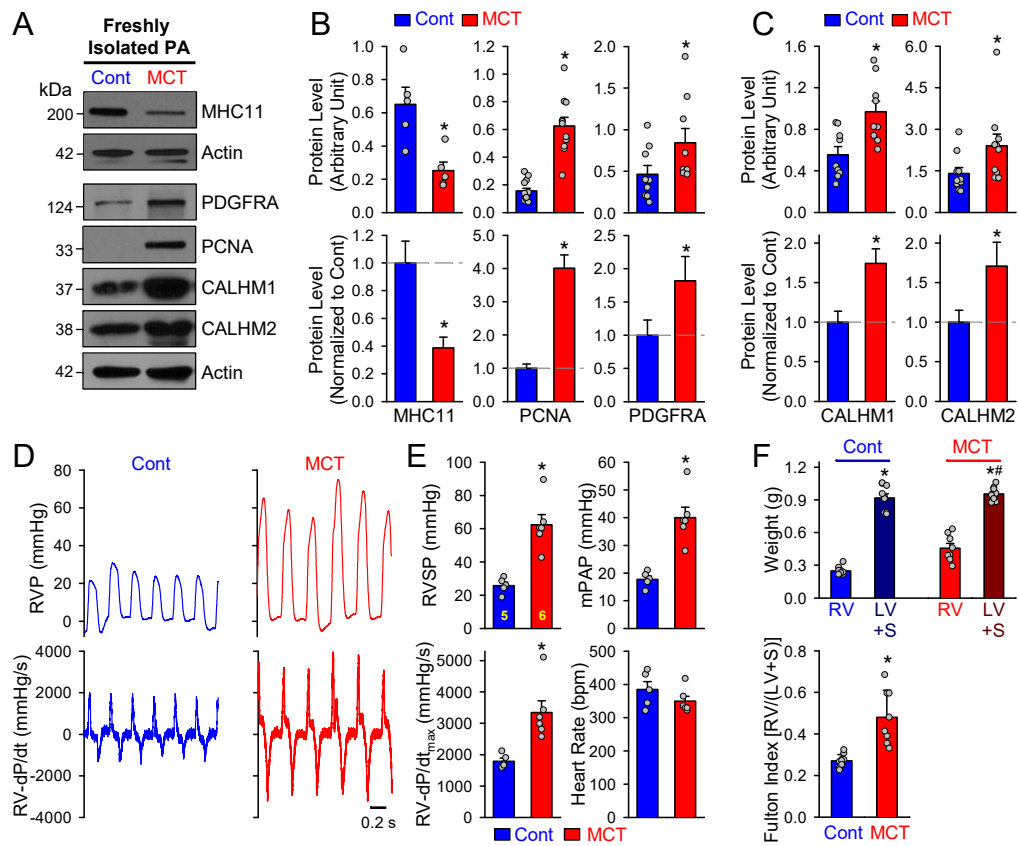

Figure 6

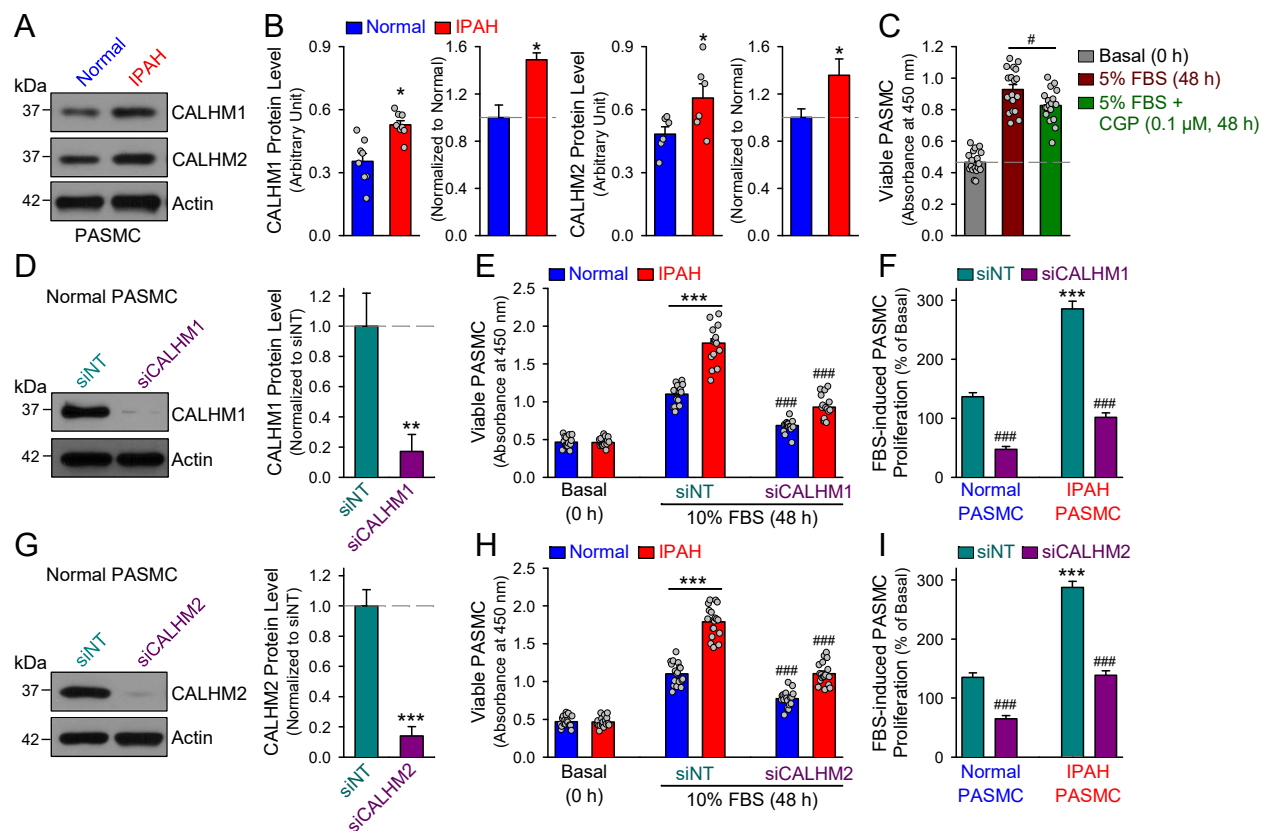

Figure 7

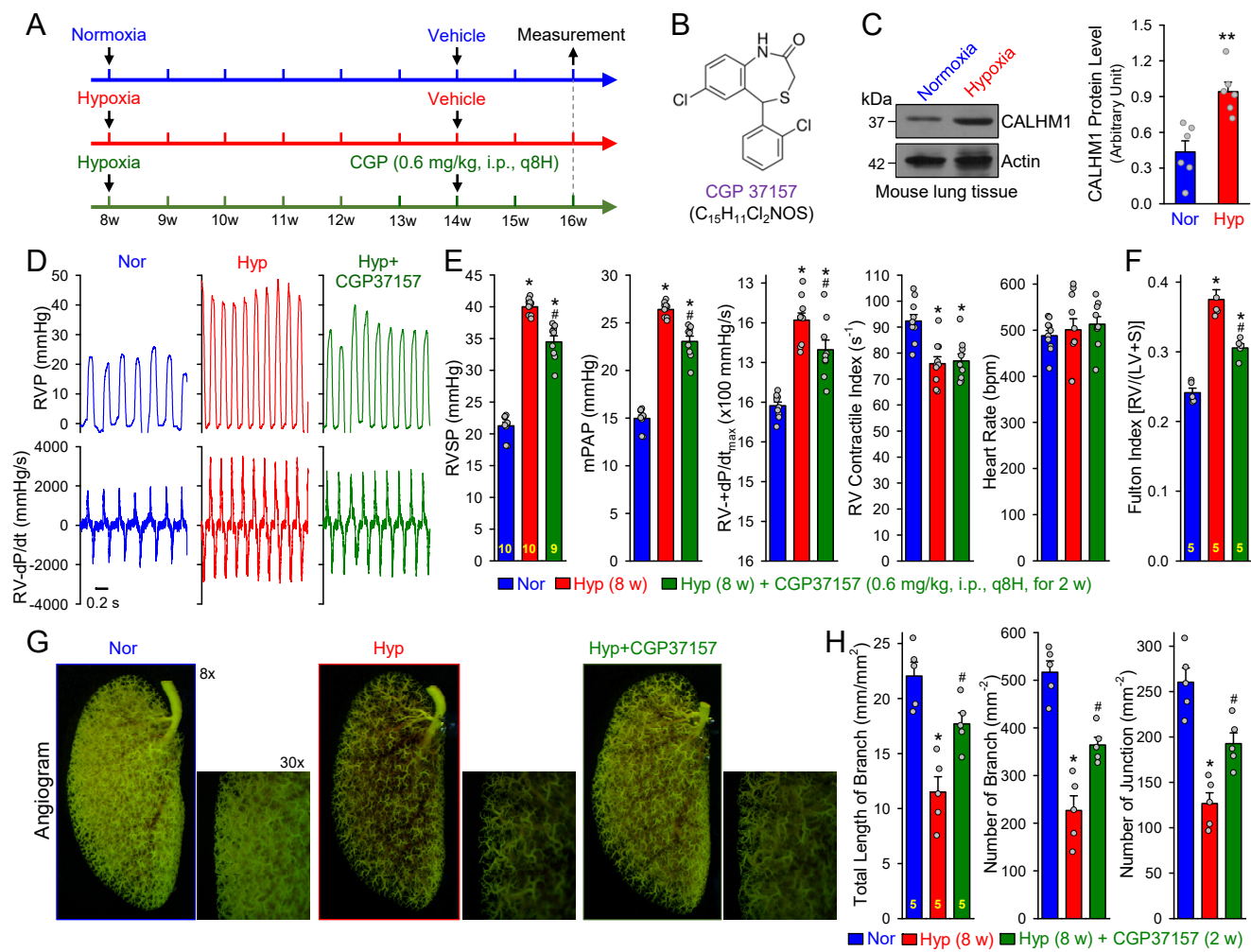

Figure 8

Supplement: Supplementary file 1 [file Data_Sheet_1.PDF]
